# Bacterial secretion of soluble and functional trivalent scFv-based N-terminal trimerbodies

**DOI:** 10.1186/s13568-015-0137-0

**Published:** 2015-08-04

**Authors:** Ana Blanco-Toribio, Ana Álvarez-Cienfuegos, Noelia Sainz-Pastor, Nekane Merino, Marta Compte, Laura Sanz, Francisco J Blanco, Luis Álvarez-Vallina

**Affiliations:** Department of Antibody Engineering, Leadartis S.L., Madrid, Spain; Molecular Immunology Unit, Hospital Universitario Puerta de Hierro, Majadahonda, Madrid, Spain; Structural Biology Unit, CIC bioGUNE, Parque Tecnológico de Bizkaia, Derio, Spain; IKERBASQUE, Basque Foundation for Science, Bilbao, Spain; Immunotherapy and Cell Engineering Laboratory, Department of Engineering, Aarhus University, Gustav Wieds Vej, 8000 Aarhus, Denmark

**Keywords:** *E. coli*, Recombinant antibody, Multivalent antibody, Trimerbody

## Abstract

Recombinant antibodies are used with great success in many different diagnostic and therapeutic applications. A variety of protein expression systems are available, but nowadays almost all therapeutic antibodies are produced in mammalian cell lines due to their complex structure and glycosylation requirements. However, production of clinical-grade antibodies in mammalian cells is very expensive and time-consuming. On the other hand, *Escherichia coli* (*E. coli)* is known to be the simplest, fastest and most cost-effective recombinant expression system, which usually achieves higher protein yields than mammalian cells. Indeed, it is one of the most popular host in the industry for the expression of recombinant proteins. In this work, a trivalent single-chain fragment variable (scFv)-based N-terminal trimerbody, specific for native laminin-111, was expressed in human embryonic kidney 293 cells and in *E. coli*. Mammalian and bacterially produced anti-laminin trimerbody molecules display comparable functional and structural properties, although importantly the yield of trimerbody expressed in *E. coli* was considerably higher than in human cells. These results demonstrated that *E. coli* is a versatile and efficient expression system for multivalent trimerbody-based molecules that is suitable for their industrial production.

## Introduction

Recombinant antibodies represent one of the fastest growing class of biopharmaceutical products and are established as important tools for research, diagnosis and therapy (Leavy 2010). First generation monoclonal antibodies (mAbs) have achieved considerable success in the treatment of a plethora of conditions including inflammation, autoimmune, cardiovascular and infectious diseases, and cancer (Reichert 2014). Next generation antibodies are designed to further increase the potency, improve the safety profile and acquire non-natural properties, and constitute a key area in the research and development of recombinant antibody (Nuñez-Prado et al. 2015). Currently, a diverse array of non-canonical antibody formats with modified architectures and additional functions has been generated (Cuesta et al. 2010; Nuñez-Prado et al. 2015). In parallel, a wide variety of production systems have been developed, ranging from bacteria (both Gram-negative and positive), yeasts, insect cell lines and mammalian cells to transgenic plants and animals (Frenzel et al. 2013).

We have recently reported the in vitro and in vivo properties of multivalent antibodies generated by fusing a trimerization (TIE) domain to the N- or C-terminus of a single-chain variable fragment (scFv) (Sánchez-Arevalo Lobo et al. 2006; Cuesta et al. 2009, 2012; Blanco-Toribio et al. 2013). TIE domains are composed of the N-terminal trimerization region of collagen XVIII NC1 (TIE^XVIII^) or collagen XV NC1 (TIE^XV^) flanked by flexible linkers. This new antibody format, termed trimerbody, is trimeric in solution and exhibited excellent antigen binding capacity and multivalency (Sánchez-Arevalo Lobo et al. 2006; Cuesta et al. 2009, 2012). Furthermore, by fusing scFv antibodies with the same or different specificity to both ends of a TIE^XVIII^ domain, we have produced monospecific or bispecific hexavalent-binding molecules, thereby expanding the scope of potential applications of trimerbody molecules (Blanco-Toribio et al. 2013). Due to their multimeric nature and the requirement of forming disulfide bonds for the generation of functional antigen-binding sites in the scFv domains, trimerbodies have been produced in eukaryotic cells until now, using yeast (*Pichia Pastoris*) and mammalian cells (Sánchez-Arevalo Lobo et al. 2006; Cuesta et al. 2009, 2012; Blanco-Toribio et al. 2013, 2014). However, nearly 30% of currently clinically approved recombinant therapeutic proteins are produced in *Escherichia coli* (Baneyx 1999; Huang et al. 2012) (Huang et al. 2012). The main reasons for this choice are cost-efficiency, well-characterized genetics, and the availability of a diverse molecular toolkit for genetic engineering (Makino et al. 2011; Overton 2014).

Here, we have studied the potential of *E. coli* for the production of scFv-based N-terminal trimerbodies using the anti-angiogenic scFv antibodies L36 and 2H1. The L36 scFv recognizes laminin-111, inhibits capillary morphogenesis of endothelial cells and prevents the establishment and growth of subcutaneous tumors in mice (Sanz et al. 2002; Sánchez-Arevalo Lobo et al. 2006), whereas the 2H1 scFv blocks the interaction between vascular endothelial growth factor (VEGF) and VEGF receptor-2 (KDR/Flk-1) and hampers endothelial cell proliferation in a dose-dependent manner (Lamdan et al. 2011). Antibody genes were cloned into the pET28a expression vector under the control of a T7 promoter and the resulting plasmids were transformed into *E. coli* BL21(DE3) cells. We have performed a comparative functional and structural analysis of the same trimerbody produced in *E. coli* or in mammalian cells, showing the functional equivalence of the two preparations. Our results demonstrate that *E. coli* is a viable alternative expression system for scFv-based N-terminal trimerbody molecules.

## Methods

### Reagents and antibodies

Mouse IgG1 Tetra-His mAb specific for His-tagged proteins was from Qiagen GmbH (Hilden, Germany). The polyclonal antibodies included: horseradish peroxidase (HRP)-conjugated goat anti-mouse IgG, Fc specific, (Sigma-Aldrich, San Louis, MO, USA), and IRDye800-conjugated donkey anti-mouse IgG (H&L) (Rockland Immunochemicals, Gilbertsville, PA, USA). Laminin-111 (LM-111) extracted from the Engelbreth-Holm-Swarm (EHS) mouse tumor was from Invitrogen Life Technologies (Carlsbad, CA, USA), bovine serum albumin (BSA) and isopropyl-1-thio-b-d-galactoside (IPTG) were from Sigma-Aldrich, and human vascular endothelial growth factor 165 (VEGF_165_) was from Peprotech (London, UK).

### Construction of expression vectors

The mammalian expression vector pCR3.1-L36-hNC1encoding the anti-LM-111 L36 scFv-based N-terminal trimerbody, containing a murine TIE^XVIII^ domain, has been previously reported (Blanco-Toribio et al. 2013). To generate the *E.coli* expression vector pET28a-L36, a 841-bp *Hin*dIII/*Not*I fragment of plasmid pHEN2-L36 (Sanz et al. 2001, 2003), containing the the pectate lyase signal peptide (*pelB*) of *Erwinia carotovora* and the L36 scFv sequence, was cloned into the *Hin*dIII/*Not*I digested backbone of plasmid pET28a (Novagen, San Diego, CA, USA). To construct the plasmid pET28a-L36-TIE, a human TIE^XVIII^ domain of was synthesized by GeneartAG (Life Technologies) and subcloned as *Not*I/*Bam*HI into the vector pET28a-L36. To generate the *E.coli* expression plasmid pET28a-2H1-TIE, the DNA fragment coding for the anti-VEGF 2H1 scFv (Lamdan et al. 2011) was synthesized by GeneArt AG and subcloned as *Sfi*I/*Not*I into the vector pET28a-L36-TIE.

### Culture conditions and expression in bacteria

*E. coli* BL21 cells [F-ompT hsdSB (rB^−^, mB^−^) gal dcm (DE3)] (Novagen) were transformed with T7 promoter driven expression vectors (pET28a). Bacteria were grown at 37°C in LB-agar plates or in liquid 2xYT medium, supplemented with appropriated antibiotics [ampicillin (Ap), 100 µg/ml; kanamycin (Km), 35 µg/ml]. For periplasm expression, 20 ml 2xYT cultures were grown overnight at 37°C under static culture conditions. The next day, the cultures were inoculated in an appropriate volume of 2xYT and incubated at 37°C and shaking at 250 rpm until OD600 = 0.5, IPTG was added to a final concentration of 0.1 mM and then cultures were incubated for 20 h at 25°C and 180 rpm. Then cultures were centrifuged at 4,000 g for 10 min at 4°C, and the pellets were resuspended in 1/20 of the initial culture volume of precooled periplasmic preparation buffer 30 mM Tris–HCl, pH 8.0, 1 mM EDTA, 20% sucrose. After a 20 min incubation on ice, bacteria were harvested by centrifugation at 6,000*g* for 10 min at 4°C. The supernatant was stored at 4°C and the pellets were resuspended in 1/20 of initial culture volume of precooled 5 mM MgSO_4_, incubated for 20 min on ice and centrifuged for 10 min at 4°C. After 20 min incubation on ice, bacteria were harvested by centrifugation at 6,000*g* for 10 min at 4°C. The two fractions were pooled, cleared by centrifugation at 30,000*g* for 20 min, and dialyzed (cut-off 10,000 Da) against PBS pH 7.4. Antibody expression was analyzed using ELISA and western blotting.

### Culture conditions and expression in mammalian cells

HEK-293 cells (CRL-1573; American Type Culture Collection, Rockville, MD, USA) were cultured in Dulbecco’s modified Eagle’s medium (DMEM) (Lonza, Walkersville, MD, USA) supplemented with 10% (vol/vol) heat inactivated fetal calf serum (FCS) (Invitrogen Life Technologies), unless otherwise stated. HEK-293 cells were transfected with the appropriate expression vectors using calcium phosphate (Compte et al. 2007). Stable cell lines were generated in HEK 293 cells and selected in DMEM with 0.5 mg/ml G-418 (Sigma-Aldrich), and the proteins were purified from conditioned medium with 0.1% (vol/vol) FCS. Antibody expression was analyzed using ELISA and western blotting.

### Protein purification

Harvested conditioned mammalian medium was centrifuged, 0.22 μm filtered (Nalgene, Neerijse, Belgium), concentrated (10×) with a 10.000 MWCO Vivaflow 50 filter (Vivascience GmbH, Hannover, Germany), dialyzed against PBS (pH 7.4) and loaded onto a HisTrap HP 1 ml column using and ÄKTA Prime plus system (GE Healthcare, Uppsala, Sweden). Dialyzed periplasmic preparations were 0.22 μm filtered and loaded onto a HisTrap HP 1 ml column using and ÄKTA Prime plus system. The purified antibodies were dialyzed against PBS, analyzed by SDS-PAGE under reducing conditions and stored at −80°C.

### Western blotting

Samples were separated under reducing conditions on 12% Tris–Glycine gels (Bio-Rad Laboratories Ltd., Hercules, CA, USA) and transferred using iBlot system (Invitrogen Life Technologies). After blocking with LI-COR blocking solution (LI-COR, Lincoln, NE, USA), proteins were detected with anti-His mAb, followed by incubation with an IRDye800 conjugated donkey anti-mouse IgG. Images were taken using the Odyssey Infrared Imaging system (LI-COR).

### ELISA

The ability of scFvs and scFv-based N-trimerbodies to bind LM-111 or VEGF_165_ was studied by ELISA as described (Cuesta et al. 2009). Briefly, Maxisorp plates (NuncA/S, Roskilde, Denmark) were coated with LM-111 (0.5 µg/well) or VEGF_165_ (0.3 µg/well) and after washing and blocking with 200 µl 5% BSA in PBS, 100 μl with indicated amount of purified protein or periplasmic extract were added for 1 h at room temperature. Antigen titration was performed with serial dilutions of purified L36 scFv-based trimerbodies, and after three washes, 100 µl of HRP-conjugated protein A (1 µg/ml) were added for 1 h at room temperature, after which the plate developed with OPD.

### Serum stability

One microgram of each purified scFv-based N-trimerbody was incubated in 60% human serum at 37°C for up to 96 h. Samples were removed for analysis at 3, 24, 48 and 96 h and frozen at −80°C until the entire study was completed. As a control, a second set of serum-exposed samples was frozen immediately to represent a zero time point. Aliquots of the different time-points samples were tested for their capability to bind LM-111 by ELISA.

### Size exclusion chromatography-multi-angle laser light scattering (SEC–MALLS)

Static light scattering experiments were performed at room temperature using a Superdex 200 G10/300 GL Size Exclusion Chromatography column (GE HealthCare, Little Chalfont, United Kingdom) attached in-line to a DAWN-HELEOS light scattering detector and an Optilab rEX differential refractive index detector (Wyatt Technology Corporation, Santa Barbara, CA, USA). The column was equilibrated with running buffer (PBS + 0.03% NaN_3_, 0.1 µm filtered) and the SEC–MALLS system was calibrated with a sample of BSA at 1 mg/ml. Then 100 µl samples of the different antibodies at 0.3 mg/ml in PBS were injected into the column at a flow rate of 0.5 ml/min. Data acquisition and analysis were carried out using ASTRA software (v 5.3.4.19, Wyatt). Based on numerous measurements on BSA samples at 1 mg/ml under the same or similar conditions we estimated that the experimental error in the molar mass is around 5%.

## Results

### Production of scFv-based N-terminal trimerbodies in the bacterial periplasm

**Fig. 1:**
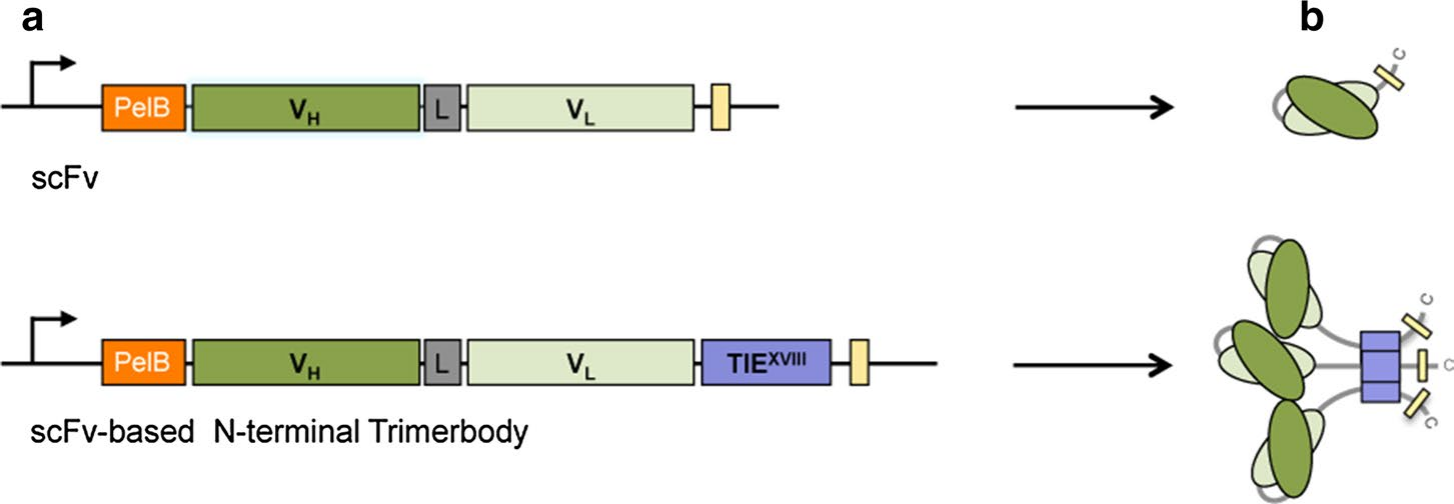
**a** Schematic diagram of the scFv and the scFv-based N-terminal trimerbody gene constructs. Both constructs carry a N-terminal pelB signal sequence for potential periplasmic localization, the anti-laminin L36 scFv gene (V_H_ and V_L_ domains joined by a flexible linker), and C-terminal tags for immunodetection and purification (*yellow box*). The trimerbody gene constructs contains a TIE^XVIII^ domain. **b** Illustration showing the arrangement of V_H_ and V_L_ domains in monomeric scFv and of V_H_, V_L_ and TIE^XVIII^ domains in the trimerbody.

**Fig. 2:**
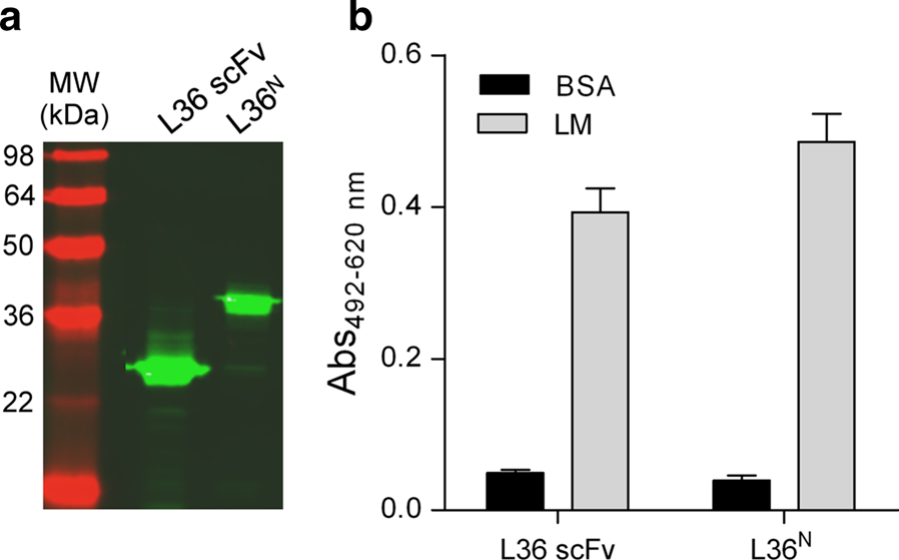
Secretion of functional L36 scFv and L36^N^ trimerbody (L36^N^) into the bacterial periplasmic. **a** Western blot profile of antibodies expressed in the periplasm of *E. coli* BL21(DE3) cells under induction with 0.1 mM IPTG. **b** ELISA against plastic immobilized laminin-111.

The L36 scFv and the L36 scFv-based N-terminal trimerbody (L36^N^) were chosen as models (Fig. 1) and cloned into plasmid pET28a which contains a T7 promoter (Studier et al. 1990). Both plasmids were transformed into *E. coli* BL21(DE3) cells. The induction of antibody expression was achieved with a combination of low temperature, reduced IPTG concentration (0.1 mM) and long induction times, and the presence and functionality of L36 scFv and L36^N^ in the periplasmic fraction was studied by western blot and ELISA. Both constructs were secreted as soluble proteins to the periplasm of *E. coli* BL21(DE3) cells (Fig. 2a). Western blot analysis, under reducing conditions, demonstrated that L36 scFv and L36^N^ trimerbody were single-chain type molecules with a migration pattern consistent with molecular weights calculated from their amino acid sequences (29.0 and 37.5 kDa, respectively) (Fig. 2a). Secreted L36 scFv and L36^N^ trimerbody specifically bound to immobilized human LM-111 (Fig. 2b).

### Purification and characterization of bacterially and mammalian-produced scFv-based N-terminal trimerbodies

**Fig. 3:**
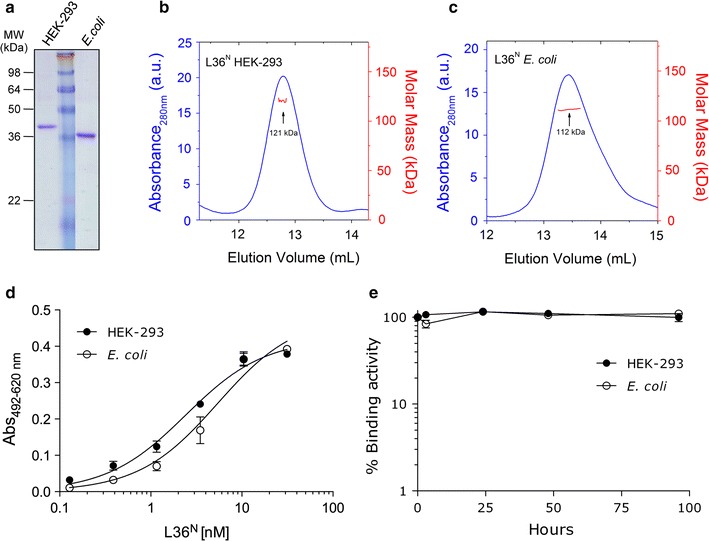
Characterization of purified L36^N^ trimerbodies. **a** Reducing SDS-PAGE of L36^N^ trimerbody purified from HEK-293 cells or *E. coli* BL21(DE3) cells. **b**, **c** Size exclusion chromatogram profile, as measured by UV absorbance at 280 nm (*blue trace*) and molar mass (*red trace*), as measured by MALLS (only the data at the central part of the peak is shown, and can be read at the *right hand axis*). The mass measured at the center of the peak is indicated. **d** The functionality of purified L36^N^ trimerbodies was demonstrated by direct ELISA with purified LM-111. Bound L36^N^ was detected using HRP-conjugated protein A. **e** Serum stability. Purified L36 ^N^ trimerbodies were incubated at 37°C for different time periods in human serum. The antigen-binding activity was analyzed by ELISA against LM-111. The experiments were performed there times, and the mean values ± standard deviations are presented.

For purification, the periplasmic fraction from transformed *E. coli* BL21(DE3) cells and the serum-free conditioned media from stably transfected HEK-293 cells were independently collected. Both L36^N^ trimerbodies were purified by immobilized metal affinity chromatography, which yielded >95% pure proteins as assessed by reducing SDS-PAGE (Fig. 3a). The production yield of mammalian-and bacterially-produced L36^N^ trimerbodies after purification were about 1 mg/l and 7 mg/l, respectively. The L36^N^ produced in *E. coli* was slightly smaller than that produced in mammalian cells, which could be attributed to differences in the lengths and amino acid compositions of the affinity tags: pCR3.1 mammalian expression plasmid contains the myc epitope and a six-histidine tag (Sánchez-Arevalo Lobo et al. 2006), whereas pET28a bacterial expression plasmid contains only a six-histidine tag.

The trimeric nature of the L36^N^ molecules was confirmed by SEC–MALLS measurements. The sample of L36^N^ from HEK-293 cells eluted from the size exclusion column as a major symmetric peak at 12.8 ml (Fig. 3b), with a small portion of high molecular weight aggregates eluting at the exclusion volume of the column. The mass derived from the dispersed light at the center of the peak is 121 kDa, a little higher than expected (117 kDa for the trimer). The sample of L36^N^ from *E. coli* produced a similar chromatogram with single peak at 13.4 ml with a measured mass of 112 kDa at its center as predicted for a trimer (Fig. 3c). Both measured molar masses are, within the experimental error, the same as the calculated values for trimeric molecules, indicating that they are indeed trimers in solution.

To avoid potential effects associated with the different tags used in both L36^N^ trimerbodies (myc-His tag in L36^N^ produced in HEK-293 and His tag in L36^N^ produced in *E. coli*), and given that the L36 scFv (V_H_3-DP47) binds to protein A (Sanz et al. 2001), LM-111 bound L36^N^ trimerbodies were developed with HRP-conjugated protein A. As shown in Fig. 3d, the binding curves of purified bacterially or mammalian-produced L36^N^ molecules to plastic immobilized human LM-111 were dose-dependent and saturable. The serum stability of both L36^N^ trimerbodies was studied by incubating each of the purified antibodies in human serum at 37°C, for prolonged periods of time. Bacterially and mammalian-produced L36^N^ molecules had similar stability, retaining more than 95% of the initial LM111-binding activity after 96 h incubation (Fig. 3e).

**Fig. 4:**
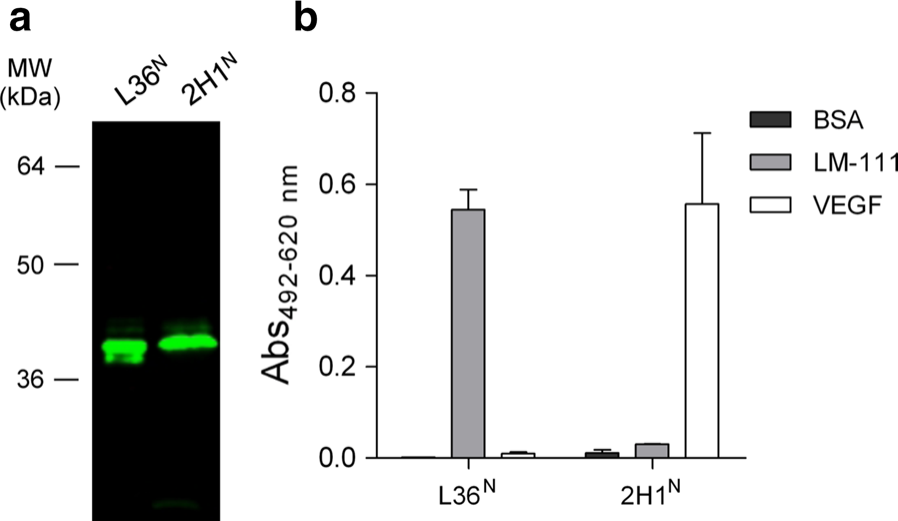
Secretion of L36 (L36^N^) and 2H1 scFv-based terminal trimerbodies (2H1^N^) in the periplasm of *E*. *coli* BL21(DE3) cells. Western blot analysis (**a**) and ELISA against plastic immobilized laminin-111 and human VEGF_165_ (**b**).

To further validate the periplasmic expression systems for the secretion of trimerbody molecules, the anti-VEGF_165_ 2H1 scFv-based N-terminal trimerbody (2H1^N^) was cloned into the pET28a plasmid. Western blot analysis under reducing conditions of periplasm extracts showed that the migration pattern of secreted L36^N^ and 2H1^N^ was consistent with the molecular weight calculated from the polypeptide sequences (Fig. 4a). Functional analysis showed that the 2H1^N^ trimerbody was able to bind specifically to immobilized human VEGF_165_ (Fig. 4b).

## Discussion

In the present study we demonstrate that functional scFv-based N-terminal trimerbody molecules are expressed and secreted in *E. coli* at significant levels (about sevenfold higher than in mammalian cells). Moreover, we demonstrate that both bacterially and mammalian-produced trimerbodies have similar functional and structural properties. Purified anti-LM-111 L36^N^ trimerbodies were trimeric in solution, as unambiguously shown by the light scattering measurements. L36^N^ trimerbodies produced in *E. coli* BL21(DE3) cells or in HEK-293 cells are both highly efficient at antigen recognition. Stability at physiological temperatures and resistance to serum proteases are critical parameters for effective therapeutic application of recombinant antibodies (Willuda et al. 1999). In this study, we have shown that bacterially-produced L36^N^ trimerbodies are remarkably stable in human serum.

*E. coli* is one of the most important production system for recombinant proteins, amenable to high cell density fermentation in bioreactors and with high yields; however, multimeric antibodies are not ideal for expression in *E. coli* as they are made from different polypeptides, which must be exported into the periplasm to be folded properly and form the appropriate disulfide bonds (de Marco 2009; Sato and Inaba 2012). An additional relevant feature is the absence of protein glycosylation machinery in prokaryotic organisms, which is important for IgG-mediated effector functions, but not for novel Fc-less engineered antibody formats, such as trimerbodies. The prediction of potential glycosylation sites using the GlycoEP server (Chauhan et al. 2013) showed that the L36^N^ and 2H1^N^ trimerbodies do not contain putative N- and O-glycosylation sites, which prompted us to investigate *E. coli*–based expression systems for the production of scFv-based trimerbodies.

In recent years, considerable effort has been devoted to the optimization of bacterial expression systems for antibody production (Gershenson and Gierasch 2011). Bacteria have been widely used in the expression of recombinant antibodies (Spadiut et al. 2014), such as full-length IgG antibodies (Mazor et al. 2007; Chan et al. 2010), scFvs (Kipriyanov et al. 1997), Fab fragments (Wiebe et al. 2010), multimeric single-domain antibodies (Wang et al. 2013), multimeric Fabs (Hutchins et al. 2011), and multimeric scFvs (Powers et al. 2012) including trivalent antibodies made by fusing the trimerizing foldon domain from bacteriophage T4 fibritin to the C-terminus of a scFv fragment (Turki et al. 2014). Several strategies have been developed for the trimerization of scFv in order to increase avidity (Nuñez-Prado et al. 2015). Trimerbodies are engineered homotrimeric antibodies based on the use of the N-terminal trimerization region of human collagen XV or XVIII NC1 domains, as trimerizing scaffolds. So far, scFv-based N-terminal trimerbodies have only been produced in eukaryotic systems (Sánchez-Arevalo Lobo et al. 2006; Blanco-Toribio et al. 2014). Here, we have demonstrated that *E. coli* may be a promising and convenient expression system to produce more affordable functional anti-angiogenic scFv-based N-terminal trimerbodies for diagnostic and therapeutic applications.
